# Evaluation zur Zufriedenheit mit und Be‑/Entlastung durch ein hausarztzentriertes Demenzversorgungsmodell: Frühe Information und Hilfen bei Demenz (FIDEM) in Göttingen

**DOI:** 10.1007/s00115-023-01557-6

**Published:** 2023-10-05

**Authors:** Iris Demmer, Michael Belz, Lea Oberbach, Eva Hummers, Jens Wiltfang, Claudia Bartels

**Affiliations:** 1 Institut für Allgemeinmedizin, Universitätsmedizin Göttingen, Göttingen, Deutschland; 2 Klinik für Psychiatrie und Psychotherapie, Universitätsmedizin Göttingen, Von-Sieboldt-Straße 5, 37075 Göttingen, Deutschland; 3 Deutsches Zentrum für Neurodegenerative Erkrankungen (DZNE), Göttingen, Deutschland; 4 Neurosciences and Signaling Group, Institute of Biomedicine (iBiMED), Department of Medical Sciences, University of Aveiro, Aveiro, Portugal

**Keywords:** Versorgungsforschung, Ambulante Versorgung, Psychosoziale Interventionen, Netzwerk, Angehörige, Healthcare research, Outpatient care, Psychosocial interventions, Networking, Caregivers

## Abstract

**Hintergrund:**

FIDEM (Frühe Informationen und Hilfen bei Demenz) ist ein sektorenübergreifendes, hausarztzentriertes Netzwerkmodell zur nachhaltigen Verbesserung der ambulanten Versorgungssituation von Demenzbetroffenen und ihren Angehörigen durch gezielte und aufsuchende Vermittlung an nichtärztliche Versorger.

**Ziel der Arbeit:**

Beschreibung der Implementierung von FIDEM in Göttingen und explorative Evaluation des Projekts hinsichtlich Zufriedenheit sowie Be‑/Entlastung der teilnehmenden Kooperationspartner (TN).

**Material und Methoden:**

FIDEM wurde 2017 in Göttingen etabliert. Quartiersbezogene Netzwerke bestanden aus hausärztlichen und nichtärztlichen Kooperationspartnern (Ergotherapeuten, Pflegeberatungsstellen, Anbieter zur Unterstützung im Alltag, ambulante Pflege, Selbsthilfe). TN wurden zum FIDEM-Vermittlungspfad geschult. Im Rahmen halbjährlicher Netzwerktreffen wurde die Evaluation des Versorgungsmodells zu o. g. Aspekten mit einem selbst entwickelten Fragebogen im Zeitraum 08/2017 bis 10/2019 durchgeführt.

**Ergebnisse:**

In sieben Netzwerken nahmen bis Oktober 2019 29 Hausarztpraxen und 46 nichtärztliche Kooperationseinrichtungen teil. In die Evaluation wurden *n* = 80 Personen (*n* = 22 TN aus Hausarztpraxen, *n* = 58 nichtärztliche TN) einbezogen. Es ergaben sich hohe Zufriedenheitswerte auf allen Skalen (M von 7,22–7,87 bei einem möglichen Maximalwert von „10“), teils höher ausgeprägt bei den TN aus Hausarztpraxen. Alle Berufsgruppen gaben eine generelle Entlastung durch die Teilnahme an, diese war bei den Hausarztpraxen signifikant stärker ausgeprägt (alle *p*-Werte < 0,001).

**Diskussion:**

FIDEM konnte außerhalb einer geförderten Modellprojektphase in Göttingen implementiert werden. Hohe Zufriedenheit und die Angabe von Entlastung sprechen für eine Fortführung mit einer vollumfänglichen Evaluation und – unter der Voraussetzung positiver Ergebnisse – für eine Verstetigung des Versorgungsmodells und einen Transfer in weitere Landkreise in Deutschland.

**Zusatzmaterial online:**

Die Online-Version dieses Beitrags (10.1007/s00115-023-01557-6) enthält zusätzliches Material (Abbildung, Tabelle). Beitrag und Zusatzmaterial stehen Ihnen auf www.springermedizin.de zur Verfügung.

## Abstract (en)

**Background:**

Early information and support in dementia (FIDEM) is a cross-sectoral, general practitioner-centered network model for the improvement of community-based care of people with dementia and their caregivers by systematically assigning them to non-physician healthcare providers.

**Objective:**

To describe the implementation of FIDEM in Göttingen, Germany and to exploratorily evaluate satisfaction and relief vs. additional workload.

**Material and methods:**

FIDEM was established in Göttingen in 2017. Community-based and district-based networks consisted of medical (general practitioners) and non-medical cooperation partners (occupational therapists, care counselling, other non-medical care providers) and were instructed to efficiently share information. During biannual network meetings from August 2017 to October 2019, a self-developed questionnaire for quality management and evaluation of the aspects of the project described above was filled out by participants attending the meetings.

**Results:**

Consecutive recruitment resulted in 7 networks by October 2019, with participation of 29 general practitioners and 46 non-medical care providers, serving as cooperation partners. Quantitative evaluation of 80 FIDEM cooperation partners revealed high satisfaction ratings with all aspects of the model (*M* from 7.22 to 7.87 out of possible „10“), with partially higher ratings on the part of primary care physicians. Furthermore, all participants reported a reduction in workload due to participation, which was significantly higher for primary care physicians across all scales (all *p*-values < 0.001).

**Conclusion:**

FIDEM has been implemented in Göttingen beyond a funded pilot project phase. High satisfaction ratings and considerable relief in workload suggest the continuation of FIDEM along with a full evaluation and, provided positive results, its consolidation of and transfer to other regions in Germany.

Demenz ist eine chronisch fortschreitende Erkrankung mit hohem Versorgungsbedarf. Hausärzte und ihre Praxisteams betreuen Menschen mit Demenz und ihre Angehörigen in allen Phasen der Erkrankung und koordinieren die multiprofessionelle ambulante Versorgung. Das Versorgungsnetzwerk Frühe Informationen und Hilfen bei Demenz (FIDEM) unterstützt Hausarztpraxen, Informationen und Hilfe zur ambulanten Versorgung von Demenzerkrankten zu erhalten, ermöglicht Austausch und Zusammenarbeit der Versorger und stellt Angebote zur Beratung, Selbsthilfe, Ergotherapie und Entlastung für Demenzbetroffene und deren Angehörige bedarfsgerecht bereit. In diesem Beitrag werden die Umsetzung von FIDEM Niedersachsen in Göttingen und Erfahrungen der Kooperationspartner vorgestellt.

## Hintergrund und Fragestellung

Für Patienten mit Demenz und ihre Angehörigen sind Hausärzte meist die primären Ansprechpartner. Die hausärztliche Betreuung und Koordination der gesundheitlichen Versorgung erfolgt hierbei oft über den gesamten Krankheitsverlauf hinweg. Dennoch ist (beginnende) Demenz eine in der Hausarztpraxis potenziell unterdiagnostizierte Erkrankung [7], obwohl dies eine wichtige Voraussetzung für den Zugang zu einer adäquaten Versorgung darstellt. Schwierigkeiten in der hausärztlichen Demenzversorgung umfassen neben einem Mangel an Zeit [6] und an demenzspezifischem Wissen [9, 11] auch Hürden bei der Differenzialdiagnostik und der Kommunikation mit Betroffenen und Angehörigen [17]. Weitere Herausforderungen stellen die Delegation zu und Zusammenarbeit mit weiteren Behandlern dar [6]. Hausärzte sehen daher einen Bedarf sowohl an Weiterbildungen zu Demenz und zur Arzt-Patient-Kommunikation als auch zu strukturierten Behandlungspfaden in der Versorgung von Demenzerkrankten und einer verbesserten Zusammenarbeit mit anderen Behandlern. Auch ein Mangel an lokalen Unterstützungsangeboten wurde von Hausärzten als potenzielle Hürde angegeben [3, 4]. Die Wirksamkeit von Interventionen für Hausärzte und ihr Praxisteam mit alleinigem Fokus auf Wissensvermittlung zur ärztlichen Versorgung von Demenzerkrankten zeigt ein heterogenes Bild: Einerseits ist ein Wissenszuwachs messbar [16], andererseits gibt es keinen positiven Effekt auf die diagnostische Qualität und das Versorgungsmanagement [8].

Hausärzte übernehmen eine wichtige Rolle in der Koordination einer multiprofessionellen sektorenübergreifenden Versorgung, die neben der Mitbehandlung durch Fachärzte auch pflegerische, ergo- und physiotherapeutische Behandlung und psychosoziale Beratung umfasst [2, 12]. So wurden in mehreren Ländern kooperative Versorgungsmodelle umgesetzt, in denen die oben genannten Gesundheitsberufe in Versorgungsteams an einer patientenzentrierten, multiprofessionellen Demenzversorgung beteiligt sind. Diese wurden hinsichtlich ihrer Akzeptanz und ihrer Auswirkung auf die Versorgungsqualität Demenzbetroffener positiv evaluiert [5, 14].

Verschiedene Projekte in Deutschland haben zum Ziel, die Versorgung Demenzkranker im hausärztlichen Versorgungsbereich mit Netzwerkkonzepten zu verbessern. So sollen bspw. im Projekt DemStepCare Krisen in der hausärztlichen Versorgung Demenzbetroffener durch ambulantes Case-Management vermieden werden [18]. Das „Leuchtturmprojekt Demenz“ des Bundesministeriums für Gesundheit förderte mehrere Projekte mit Schwerpunkt auf der Etablierung regionaler Versorgungnetzwerke, deren Fokus auf einer stärkeren Einbindung nichtärztlicher Unterstützungsangebote in der Demenzbehandlung lag [13]. Hierbei wurde eine positive Bilanz hinsichtlich der Beteiligung von Hausärzten und medizinischen Fachangestellten (MFA) gezogen [15].

FIDEM (Frühe Informationen und Hilfen bei Demenz) ist ein Netzwerkkonzept, welches – ausgehend von der Landesvereinigung für Gesundheit und Akademie für Sozialmedizin Niedersachsen e. V. (LVG & AFS) als Projektträgerin – 2009–2012 (FIDEM I: Region Braunschweig) und 2013–2016 in mehreren Städten und Landkreisen in Niedersachsen (FIDEM II: Osterode am Harz, Lüneburg, Grafschaft Bentheim) als Modellprojekt in Kooperation mit der ambulanten Betreuung hilfe- und pflegebedürftiger Menschen (ambet) e. V. Braunschweig erprobt und durch die Hochschule Osnabrück qualitativ evaluiert wurde [1]. Als Modellprojekte wurden FIDEM I und II vom Land Niedersachsen, den gesetzlichen Pflegekassen und den privaten Pflegeversicherungsunternehmen gefördert. Anschließend erfolgte die Freigabe des Konzepts niedersachsenweit (ohne Förderung).

Als übergeordnetes Ziel verfolgt FIDEM die frühzeitige, sektorenübergreifende, strukturierte und wohnortnahe Verbesserung der Versorgungssituation von Demenzbetroffenen und ihren Angehörigen. Weiterhin sollen Hausarztpraxen entlastet sowie eine Erhöhung der Bekanntheit und der bedarfsgerechten Zuweisung zu nichtärztlichen Versorgungsangeboten angestrebt werden. In der hier vorliegenden explorativen Evaluation des Transfers des Versorgungsmodells FIDEM Niedersachsen nach Göttingen sollte untersucht werden,

1. inwieweit eine Umsetzung gelang,
2. wie die Kooperationspartner ihre eigene Zufriedenheit mit dem Netzwerkprojekt einschätzten und
3. inwieweit eine Be- oder Entlastung der verschiedenen Berufsgruppen (ärztliche vs. nichtärztliche Kooperationspartner) durch eine Teilnahme erreicht wurde.

## Methodik

### Beschreibung des Versorgungs- und Kooperationsmodells FIDEM Göttingen

**Figure 1:**
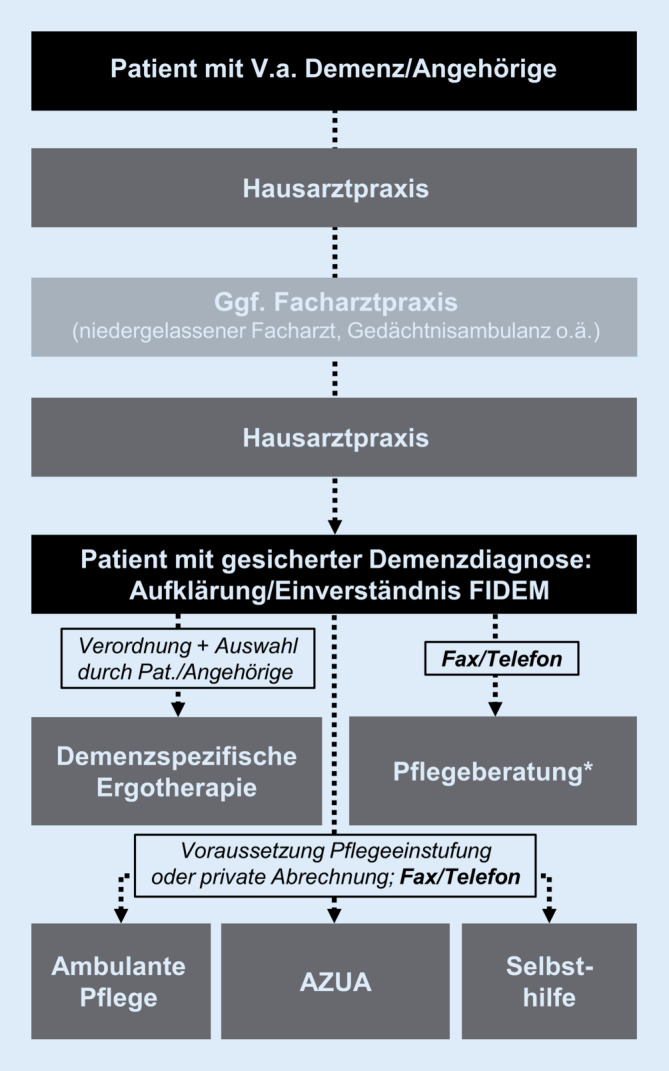

Das Versorgungs- und Kooperationsmodell FIDEM soll die sektorenübergreifende Vernetzung nichtärztlicher Unterstützungsangebote mit der hausärztlichen Versorgung fördern. Nach optionaler fachärztlicher Sicherung einer Demenzdiagnose (möglichst in einem frühen Stadium) sollten Patienten in der Hausarztpraxis über die zugehende Vermittlung an nichtärztliche Beratungs- und Behandlungsangebote informiert werden. Die Diagnose erfolgte in hausärztlicher Regie leitliniengerecht, d. h. fach- und/oder hausärztlich gemäß AWMF-Leitlinie Demenzen. Sie wurde nicht zusätzlich durch eine zentrale Stelle hinsichtlich Güte oder Schweregrad geprüft. Nach schriftlicher Einwilligung und Schweigepflichtentbindung gegenüber den FIDEM-Kooperationspartnern konnten Patienten und Angehörige per FIDEM-Faxformular direkt und quartiersnah an nichtärztliche Kooperationspartner vermittelt werden^1^. Diese vereinbarten mit den Betroffenen individuelle Maßnahmen (aufsuchendes/zugehendes Versorgungsangebot) und gaben über das Faxformular Rückmeldung an die auftragserteilenden Hausarztpraxen zu durchgeführten Maßnahmen (Abb. 1). Im konkreten Fall erfolgte üblicherweise zunächst eine Faxanforderung an eine der Pflegeberatungsstellen. Mit einem zugehenden/aufsuchenden Vorgehen wurde der Betroffene zu einer Terminvereinbarung kontaktiert und erhielt eine Pflegeberatung (u. a. zur Stellung eines Antrags auf Pflegeleistungen). Von dort erfolgte nach individueller Analyse des Hilfebedarfs eine Weitervermittlung an nachfolgende Versorger (z. B. Laienhilfsdienste, Betreuungsgruppen, Tagesbetreuung, Tagespflege, Selbsthilfe- oder Angehörigengruppen), die über die Faxvermittlung ebenfalls zugehend vorgingen. Parallel dazu erfolgte eine Verordnung von Ergotherapie sowie der analog gestaltete Einbezug von an FIDEM-beteiligten Ergotherapiepraxen. Zur Rückmeldung des aktuellen Versorgungsstandes wurde von den involvierten Parteien ebenfalls der Faxkommunikationsweg genutzt. Ein wechselseitiger, informeller Austausch zwischen den beteiligten Behandlern war zusätzlich möglich. Es fanden halbjährliche Netzwerktreffen der Kooperationspartner statt.

**Figure 2:**
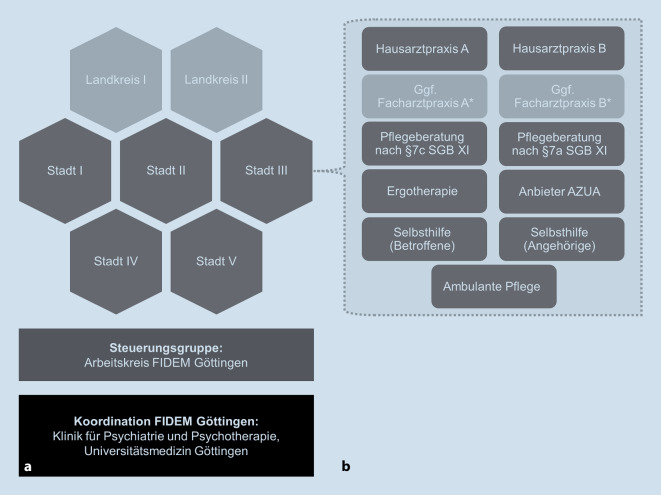

Ausgehend von den Vorgängermodellprojekten FIDEM I (2009–12) und II (2013–16) wurde FIDEM zur Umsetzung im Landkreis und der Stadt Göttingen weiterentwickelt und modifiziert: (1) Die Koordination erfolgte nicht durch die ortsansässigen Senioren- und Pflegestützpunkte wie in den Vorgängermodellen, sondern oblag der Klinik für Psychiatrie und Psychotherapie der Universitätsmedizin Göttingen (UMG), vertreten durch die Projektleiterin und beraten durch eine Steuerungsgruppe (Arbeitskreis FIDEM Göttingen) mit regionalen Vertretern aus allen beteiligten Berufsgruppen. (2) Individuelle Schulungen für Hausärzte und MFA fanden in der eigenen Praxis statt (vorher: zentral, getrennt für Hausärzte und MFA). (3) Als ergotherapeutische Kooperationspartner geeignet waren solche mit Erfahrung in der Demenzbehandlung (Zertifizierung für demenzspezifische Ergotherapie nicht obligat; siehe Abb. 2 für ein Schema zur Organisations- und Netzwerkstruktur von FIDEM Göttingen).

Kooperationspartner (hausärztlich und nichtärztlich) wurden konsekutiv seit 21.06.2017 zur Teilnahme über öffentlich zugängliche Datenbanken sowie durch eine Informationsveranstaltung, Öffentlichkeitsarbeit, Ansprache in Qualitätszirkeln und Netzwerken (z. B. Arbeitskreis Gerontopsychiatrie des Sozialpsychiatrischen Verbundes Göttingen), schriftliche und telefonische Einladungen und Mund-zu-Mund-Propaganda rekrutiert und zum FIDEM-spezifischen Vermittlungspfad geschult. Hausarztpraxen erhielten zusätzlich zu Beginn durch die Koordinatorin eine 1‑ bis 2‑stündige Schulung in der eigenen Praxis und wurden mit demenz- sowie projektspezifischem Material ausgestattet (u. a. Screeningtests, demenz- und FIDEM-spezifische Informationen, Kontaktadressen, Flyer, Broschüren).

### Explorative Evaluation des Projekts

Es wurde eine prospektive und explorative Evaluation innerhalb eines Versorgungsbegleitforschungsansatzes des Versorgungs- und Kooperationsmodell FIDEM in der Region Göttingen anhand von Fragebögen durchgeführt. Ziele dieser Evaluation waren – neben einer Quantifizierung der Zufriedenheit sowie der Be‑/Entlastung (siehe Abschnitt Messinstrument) – die Qualitätssicherung und Ableitung optimierender Maßnahmen. Die Befragung der Kooperationspartner erfolgte nach schriftlicher und mündlicher Information sowie impliziter Einwilligung wiederholt im Rahmen der zweimal jährlich stattfindenden Netzwerktreffen in anonymisierter Form. Ein anonymes longitudinales Monitoring der teilnehmenden Kooperationspartner (TN) wurde durch einen selbst definierten Code ermöglicht, der keine direkten Rückschlüsse auf die konkrete Person zuließ. Es wurden keine Daten erhoben, die einen Rückschluss auf einzelne Patienten ermöglichten.

Die Fragebogenevaluation wurde der Ethikkommission der UMG vorgelegt (#21/2/21). Die Datenerhebung erfolgte im Zeitraum vom 15.08.2017 bis zum 23.10.2019.

### Messinstrument

Aufgrund der fehlenden inhaltlichen Passung etablierter psychometrischer Instrumente mit den projektbezogenen Messgrößen und der intendierten Fragestellung wurden Fragebogenitems selbst entwickelt. Neben allgemeinen Angaben (u. a. Praxisform und -lage, Versorgungsgruppe: TN aus Hausarztpraxen vs. nichtärztliche TN) wurden die in dieser Evaluation ausgewerteten numerischen Ratings zur (1) *Zufriedenheit mit FIDEM* (5 Items) sowie zur (2) zur *Be- und Entlastung durch FIDEM* (4 Items) erfasst. Weiterhin wurde die von FIDEM unabhängige *allgemeine Arbeitszufriedenheit* (7 Items, Kontrollvariable) erfasst. Angaben zur Zufriedenheit erfolgten auf einer 11-stufigen numerischen Skala („*0* *=* *keine Zufriedenheit“* bis „*10* *=* *maximale Zufriedenheit“*), zur Be- und Entlastung auf einer 11-stufigen, bipolaren numerischen Skala (*„−5* *=* *extreme Belastung“* bis *„+5* *=* *extreme Entlastung“*; siehe Online-Supplement eTab. 1 für diese Items).

### Statistische Auswertung

Für die Datenanalyse wurde die Software IBM SPSS Statistics (Version 29) (IBM Corporation, Armonk, NY, USA) verwendet. Zur deskriptiven Darstellung der Variablen erfolgte die Berechnung von Häufigkeiten (Frequenzen), prozentualer Verteilung (%) und Mittelwerten mit Standardabweichungen (M ± SD).

Es wurden zwei primäre Zielgrößen definiert: (1) *Zufriedenheit mit FIDEM* (5 Items), (2) *Be- und Entlastung durch FIDEM* (4 Items). Für beide Zielgrößen wurde jeweils ein allgemeines lineares Modell für messwiederholte Daten (GLM) berechnet. Die numerischen Ratings der TN wurden als 4‑ bzw. 5‑stufiger Innersubjektfaktor in das jeweilige Modell integriert. Die Integration der Versorgungsgruppe „TN aus Hausarztpraxen“ vs. „nichtärztliche TN“ erfolgte als 2‑stufiger Zwischensubjektfaktor. Weiterhin wurde der Interaktionseffekt (Versorgungsgruppe × Ratings) in beiden GLM auf Signifikanz getestet. Die zugehörigen *p*-Werte wurden aufgrund der α‑Fehler-Inflation innerhalb jedes GLM nach Bonferroni korrigiert (initiales Signifikanzniveau: α = 0,05, zweiseitig). Neben den Haupteffekten erfolgte die Testung auf Unterschiede zwischen den einzelnen Items mittels explorativer, nichtkorrigierter Post-hoc-Tests (t-Tests) mit Effektstärken nach Cohen (*d*). Ein analoges Vorgehen mit eigenständigem GLM erfolgte für die Kontrollvariable zur *allgemeinen *(von FIDEM unabhängigen) *Arbeitszufriedenheit*. Den TN stand es frei, einzelne Items des Fragebogens nicht zu beantworten, daher variierte die Anzahl an eingeschlossenen Fällen (t-Tests: gültige Fälle = *df* + 2; GLM: siehe Angabe).

## Ergebnisse

### Implementierung von FIDEM in Göttingen

In der Stadt und im Landkreis Göttingen wurden am 21.06.2017 im Rahmen einer Auftaktveranstaltung sieben quartiersbezogene Netzwerke gegründet, bestehend aus Hausarztpraxen, Pflegeberatungsstellen, Ergotherapeuten und Anbietern von Unterstützungs- und Entlastungsangeboten, ambulanter Pflege und Selbsthilfe. Es fanden im Zeitraum vom 15.08.2017 bis zum 23.10.2019 in ca. halbjährlichen Abständen 35 Netzwerktreffen (je 5 pro Netzwerk) statt (anschließend pandemiebedingtes Aussetzen bis Herbst 2022). Zum Zeitpunkt der letzten Netzwerktreffen (Herbst 2019) nahmen 75 kooperierende Einrichtungen an FIDEM Göttingen teil, 29 Hausarztpraxen (mit 42 Hausärzten, einer Teilnahmequote von 16,4 % entsprechend bei ca. 176 Hausarztpraxen im Gebiet) und 46 nichtärztliche Einrichtungen (siehe auch Online-Supplement eAbb. 1 zur quartalsweisen Entwicklung der TN-Zahlen von Hausarztpraxen). Dabei verteilten sich 19 Hausarztpraxen auf den Landkreis, 10 auf das Stadtgebiet. Nichtärztliche Kooperationspartner versorgten in der Regel überörtlich.

### Stichprobe der Fragebogenevaluation FIDEM Göttingen

Die Mehrheit der FIDEM-Kooperationspartner nahm an jeweils einem einzelnen Netzwerktreffen mit explorativer Evaluation zur Zufriedenheit und Be‑/Entlastung teil. Bei mehrmaligen Teilnahmen wurde die letzte Messung in die Wertung aufgenommen, da hier die längste Erfahrung mit dem Projekt gegeben war.

Von insgesamt *N* = 80 Personen waren *n* = 58 nichtärztliche TN (72,5 %), *n* = 22 (27,5 %) TN stammten aus Hausarztpraxen (Praxissitz: Stadt, *n* = 15, 68,2 %; Landkreis, *n* = 7, 31,8 %). Weitere Angaben zu TN aus Hausarztpraxen finden sich in Tab. 1. Zur Gewährleistung der Anonymität der Umfrage wurde auf die Erhebung weiterer soziodemographischer Daten verzichtet. Schätzungen zur Anzahl von Patienten konnten von nichtärztlichen TN (Angestellte in den Einrichtungen) nicht plausibel vorgenommen werden.

**Table 1:** 

Angaben zu teilnehmenden Hausarztpraxen	*n* (%) bzw. M/SD
*Praxissitz*	
Stadtgebiet	15 (68,2)
Landkreis	07 (31,8)
*Praxisform*	
Gemeinschaftspraxis	10 (45,5)
Einzelpraxis	08 (36,4)
Praxisgemeinschaft	04 (18,2)
*Anzahl Patienten pro Quartal (Schätzung)*	1325/570
*Anzahl Demenzpatienten pro Quartal (Schätzung)*	34,71/25,72
*Anzahl vermuteter weiterer Demenzpatienten pro Quartal (Schätzung)*	27,52/24,67

*M* Mittelwert, *SD* Standardabweichung

### Zufriedenheit mit FIDEM Göttingen

Die Zufriedenheit mit FIDEM war auf der Skala von 0 bis 10 über beide Versorgungsgruppen hinweg für alle 5 Items mit Werten > 7 ausgeprägt (*M* = 7,22–7,87, SD 1,84–2,46). Innerhalb des GLM variierte die Zufriedenheit für die Gesamtstichprobe signifikant (Innersubjekteffekt: *F* [4, 232] = 7,71, *p* < 0,001, partielles η^2^ = 0,12; gültige Fälle: *n* = 60 von 80). Am stärksten war die „Zufriedenheit mit dem Projekt im Allgemeinen“ ausgeprägt (M = 7,82, SD = 1,91), am schwächsten die „Zufriedenheit mit der eigenen Beteiligung“ (M = 7,29, SD = 2,11). Hierbei war der Unterschied zwischen der „Zufriedenheit mit der eigenen Beteiligung“ und allen anderen Dimensionen statistisch signifikant (*p* = 0,043 bis < 0,001; alle weiteren ns).

**Figure 3:**
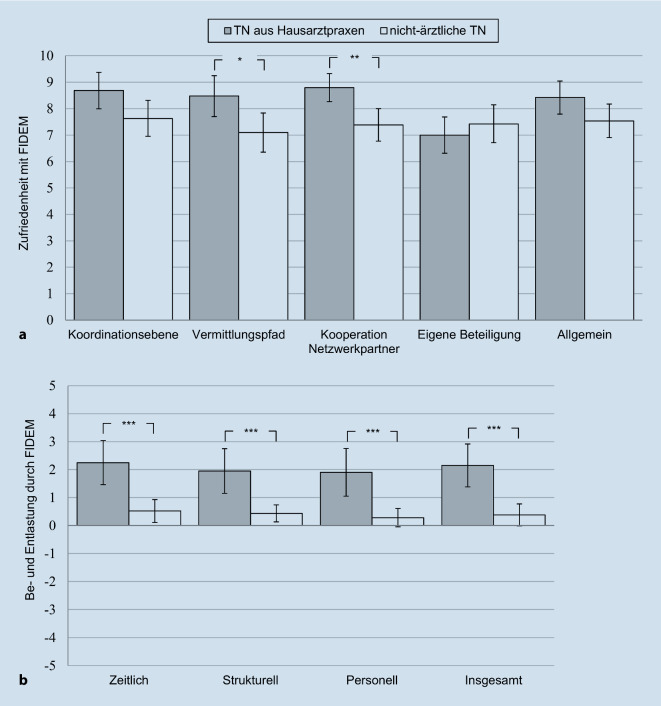

Bezogen auf Unterschiede zwischen den Versorgungsgruppen zeigte sich, dass die TN aus Hausarztpraxen in 4 von 5 Items höhere Werte erzielten als die nichtärztlichen TN (Abb. 3a). Der Zwischengruppeneffekt verfehlte die Signifikanz (*F* [1, 258] = 2,90, *p* = 0,094, partielles η^2^ = 0,05), jedoch konnte ein signifikanter Interaktionseffekt gefunden werden (*F* [4, 232] = 7,45, *p* < 0,001, partielles η^2^ = 0,11). Paarweise Vergleiche zeigten signifikant höhere Zufriedenheitswerte der TN aus Hausarztpraxen bei den Items „Vermittlungspfad“ (*t* [61] = 2,21, *p* = 0,031, *d* = 0,61) und „Kooperation mit Netzwerkpartnern“ (*t* [63] = 2,77, *p* = 0,007, *d* = 0,74; Abb. 3a).

Weiterhin wurde die Kontrollvariable der FIDEM-unabhängigen „allgemeinen Arbeitszufriedenheit“ (7 Items) analysiert, um eine insgesamt positiv oder negativ verzerrende Antworttendenz einer der Versorgungsgruppen auszuschließen. Hierbei konnten weder eine Variation der Items für die Gesamtstichprobe (Innersubjekteffekt: *F* [6, 426] = 0,50, ns; gültige Fälle: *n* = 73 von 80) noch signifikante Unterschiede zwischen den Versorgungsgruppen gefunden werden (Zwischengruppeneffekt: *F* [1, 71] = 0,01, ns; Interaktionseffekt: *F* [6, 426] = 1,45, ns; alle paarweisen Vergleiche ns).

### Be- und Entlastung durch FIDEM Göttingen

Die Be- und Entlastung wurde über beide Versorgungsgruppen hinweg auf der Skala von −5 bis +5 für alle 4 Items (zeitlich, strukturell, personell, insgesamt; siehe auch Online-Supplement eTab 1 für Itemformulierungen) im entlastenden Bereich > 0 angegeben (M = 0,90–1,11, SD 1,51–1,72). Es konnte keine signifikante Variation zwischen den 4 Items für die Gesamtstichprobe gefunden werden (Innersubjekteffekt: *F* [3, 192] = 1,41, ns, alle paarweisen Vergleiche ns; gültige Fälle: *n* = 66 von 80).

Es ergaben sich Unterschiede zwischen den Versorgungsgruppen: TN aus Hausarztpraxen bewerteten die Entlastung durch FIDEM in allen 4 Items stärker als nichtärztliche TN (Abb. 3b; Zwischengruppeneffekt: *F* [1, 64] = 23,49, *p* < 0,001, partielles η^2^ = 0,27), während der Interaktionseffekt durch das gefundene konstante Muster keine Signifikanz erreichte (*F* [3, 192] = 0,30, *ns*). Paarweise Vergleiche zeigten signifikant höhere Entlastung bei den TN aus Hausarztpraxen bei allen 4 Items (*df* = 65–68, *t* = 3,81–4,52, alle *p*-Werte < 0,001, *d* = 1,01–1,21; Abb. 3b). Hier sei darauf hingewiesen, dass die nichtärztlichen TN für kein Item den Wert „0“ unterschritten und sich damit insgesamt trotz der Unterschiede zu den TN aus Hausarztpraxen ebenfalls im „entlastenden“ Bereich befanden (*M* = 0,37–0,66, SD = 1,14–1,48; Abb. 3b).

## Diskussion

Das ambulante Demenzversorgungskonzept FIDEM konnte aus einer Modellprojektphase (FIDEM I/II) nach Göttingen übertragen und mit 75 kooperierenden Einrichtungen erfolgreich in deren Versorgungsalltag integriert werden. In der explorativen Evaluation gaben zudem sowohl ärztliche und nichtärztlichen TN eine hohe Zufriedenheit an. Eine Entlastung bei der Umsetzung der Versorgungsaufgaben wurde insbesondere durch ärztliche TN angegeben.

FIDEM Göttingen wurde mit 29 von ca. 176 Hausarztpraxen im Gebiet umgesetzt (Teilnahmequote: 16,4 %) – dies weist auf Teilnahmebarrieren hin. Potenzielle Ursachen sind sowohl mangelnde zeitliche Ressourcen als auch fachliche Unsicherheiten in Diagnostik und Behandlung der Erkrankung sowie der Kommunikation mit den Betroffenen und ihren Angehörigen [9, 17]. Auch ein wahrgenommenes Kenntnisdefizit über regionale Beratungsangebote und Behandlungspfade sowie Versorgungstrukturen zur multiprofessionellen, sektorenübergreifenden Versorgung könnte paradoxerweise eine Rolle spielen [17].

Gleichzeitig stellt sich die Rekrutierung von Hausärzten für Studien generell als herausfordernd dar [10]. So waren in dem innovationsfondsgeförderten Projekt DemStepCare Hausärzte schwer zur Teilnahme zu motivieren, da sie den unmittelbaren Nutzen für ihre Versorgungstätigkeit nur im Interventionsarm der Studie sahen. Auch fiel es ihnen schwer, sich gegenüber innovativen Versorgungsformen, wissenschaftlichen Konzepten und neuen Strukturen zu öffnen [18]. Die in der hier vorliegenden explorativen Evaluation gefundenen hohen Zufriedenheitswerte und Entlastung hinsichtlich der Versorgungsaufgaben durch FIDEM sollten bei zukünftiger Ansprache von Hausärzten daher argumentativ herangezogen werden, um die Teilnahmebereitschaft zu erhöhen. Auch sollte dahingehend differenziert werden, dass es sich bei FIDEM nicht vorrangig um eine Studie, sondern um ein unterstützendes Versorgungsmodell handelt, von dem alle Beteiligten (Patienten, Angehörige und ihre Versorger) profitieren können.

Im Vergleich zu den FIDEM-Vorgängermodellen mit 19 bzw. 16 teilnehmenden Hausarztpraxen in mehreren Landkreisen (FIDEM I bzw. II) ist die Teilnahmequote insgesamt als zufriedenstellend zu bewerten, zumal die Umsetzung von FIDEM Göttingen im Gegensatz zu den FIDEM-Modellprojekten bislang ohne Fördermittel erfolgte. Es ist anzunehmen, dass die diversen Strategien der Ansprache potenzieller Teilnehmer, die an der UMG angesiedelte Projektkoordination und eine transparente und umfassende Vermittlung von Informationen zu Zielen und Vorgehensweisen in den Praxen als auch das hohe Engagement der Projektteilnehmer zur Etablierung des Netzwerkes beigetragen haben. Eine Ausweitung und Verstetigung dieses Demenzversorgungsmodells könnte perspektivisch mit Fördermitteln für die koordinierenden Mitarbeiter, Sachmittel und Netzwerkarbeit gesichert werden.

## Limitationen

Die vorliegende Untersuchung umfasste nicht die direkten Auswirkungen auf die Versorgungssituation Demenzbetroffener und ihre Angehörigen durch FIDEM, sondern diente dazu, den Implementierungserfolg – im Besonderen ausgedrückt als Zufriedenheit mit und Be‑/Entlastung durch FIDEM – exklusiv auf Seiten der Kooperationspartner i. S. einer Versorgungsbegleitforschung explorativ zu evaluieren (Behandlerperspektive). Implizit ist eine Wirkung einzelner psychosozialer Interventionen für Demenzbetroffene anzunehmen, zumal dafür bereits eine Vielzahl an Studien vorliegt (AWMF-S3-Leitlinie Demenzen).

Von Seiten der Behandler basierte die Untersuchung ausschließlich auf Angaben teilnehmender Kooperationspartner, somit ist ein Selektionsbias nicht auszuschließen. Einstellungen nichtteilnehmender Versorger wurden nicht systematisch erfasst; dies sollte in zukünftigen Studien zur Erhöhung des Rekrutierungserfolgs umgesetzt werden. Bei der Stichprobe der explorativen Fragebogenevaluation handelt es sich zudem um eine Subgruppe an Kooperationspartnern, begründet durch die Datenerhebung i. R. der Netzwerktreffen. Ein Antwortbias ist trotz der Anonymisierung nicht vollständig auszuschließen: Die Anwesenheit auf den Netzwerktreffen kann zu einem stärkeren Benefit durch FIDEM und somit zu positiveren Einschätzungen geführt haben. Gleichzeitig war dieser Benefit jedoch auch das Ziel des Versorgungsprojekts und könnte für eine Teilnahme an ebendiesem sprechen. Eine longitudinale Evaluation war durch die häufig nur einfache Teilnahme der Kooperationspartner an den einzelnen Treffen nicht umsetzbar.

Eine Einbindung niedergelassener Fachspezialisten wurde in FIDEM Göttingen nicht erreicht. Vereinzelt erfolgten Rückmeldungen, dass diese bereits ausreichend vernetzt seien. Eine fachärztliche-psychiatrische Expertise war jedoch durch die Koordination der Klinik für Psychiatrie und Psychotherapie an der UMG gegeben.

Inhaltlich wurden als primäre Zielgrößen die Zufriedenheit mit und die Be‑/Entlastung durch eine Beteiligung an FIDEM Göttingen aus Behandlerperspektive definiert. Zur Ableitung optimierender Maßnahmen für die konkrete Umsetzung liegen Freitextantworten vor. Hier forderten TN in der Gesamttendenz eine Projektausweitung/-intensivierung („data not shown“). Auch wenn bei einzelnen TN eine geringe bzw. nicht sichtbare Aktivität im Projekt erfolgte, wurde in keinem Fall eine Teilnahme zurückgezogen, was potenziell ebenfalls für die Beibehaltung des Projekts spricht. Die Möglichkeit zur Freitextantwort und damit zur weiteren Ausgestaltung wurde jedoch insgesamt selten genutzt und bedarf zukünftig weiterer Untersuchung.

Insgesamt können die Erfahrungen und Erkenntnisse aus dieser explorativen Evaluation genutzt werden, um weiter zur Sensibilisierung für ambulante Versorgungswege sowie zur Verbesserung der Versorgungssituation für Demenzbetroffene und deren Angehörige bereits in einem früheren Stadium beizutragen. Eine nachfolgende, umfassendere Gesamtevaluation des Projekts mit einem aufwendigeren Untersuchungsdesign, einschließlich einer Kontrollgruppe (z. B. „treatment as usual“), sozioökonomischer Analysen und patientenrelevanter Outcomes kann zusätzlich die Sichtbarkeit des Projektes FIDEM auch überregional erhöhen und dessen weitere Nachhaltigkeit über einen Modellprojektstatus hinaus argumentativ unterstützen.

## Fazit für die Praxis

- Das hausarztzentrierte Versorgungskonzept FIDEM Niedersachsen (Frühe Informationen und Hilfen bei Demenz) konnte erfolgreich in Göttingen etabliert werden und wurde zur bedarfsgerechten Vermittlung von Demenzbetroffenen und ihren Angehörigen an weiterführende Versorgungsangebote genutzt.
- Hausärztliche und nichtärztliche Kooperationspartner gaben in der explorativen Evaluation eine hohe Zufriedenheit und eine Entlastung durch ihre Teilnahme an FIDEM an.
- Diese ersten quantitativen Ergebnisse sprechen für eine nachhaltige Verstetigung und Ausweitung des Versorgungskonzepts mit Gewinnung neuer Netzwerkpartner und einem überregionalen Transfer sowie für die Notwendigkeit einer kontinuierlichen (Landes‑)Förderung.
- Damit verbunden ist perspektivisch eine umfassendere Gesamtevaluation zu gesundheitsökonomischen Aspekten, die ebenfalls um die Sichtweise der Versorgungsempfänger im Vergleich zur regulären Versorgung erweitert werden sollte.

### Supplementary Information






## Footnotes

1 Demenzspezifische Ergotherapie per Verordnung, Pflegeberatung der Krankenkassen nach SGB XI § 7a oder der Pflegestützpunkte nach SGB XI § 7c, Angebote zur Unterstützung im Alltag (AZUA), ambulante Pflege, Selbsthilfegruppen für Betroffene und/oder Angehörige.
