# Supplementary material for: Evaluation of satisfaction with and relief vs. workload by a general practitioner-centered dementia care project: early information and support in dementia (FIDEM) in Göttingen, Germany
Source: Nervenarzt. 2023 Oct 5;94(11):1034–42. [Article in German] doi: 10.1007/s00115-023-01557-6 (PMC10620282; doi:10.1007/s00115-023-01557-6)
Supplement: Supplementary file 1 [file 115_2023_1557_MOESM1_ESM.docx]

**Online Supplement**

**eAbb. 1:** Quartalsweise Entwicklung der FIDEM-Teilnehmerzahlen von Hausarztpraxen


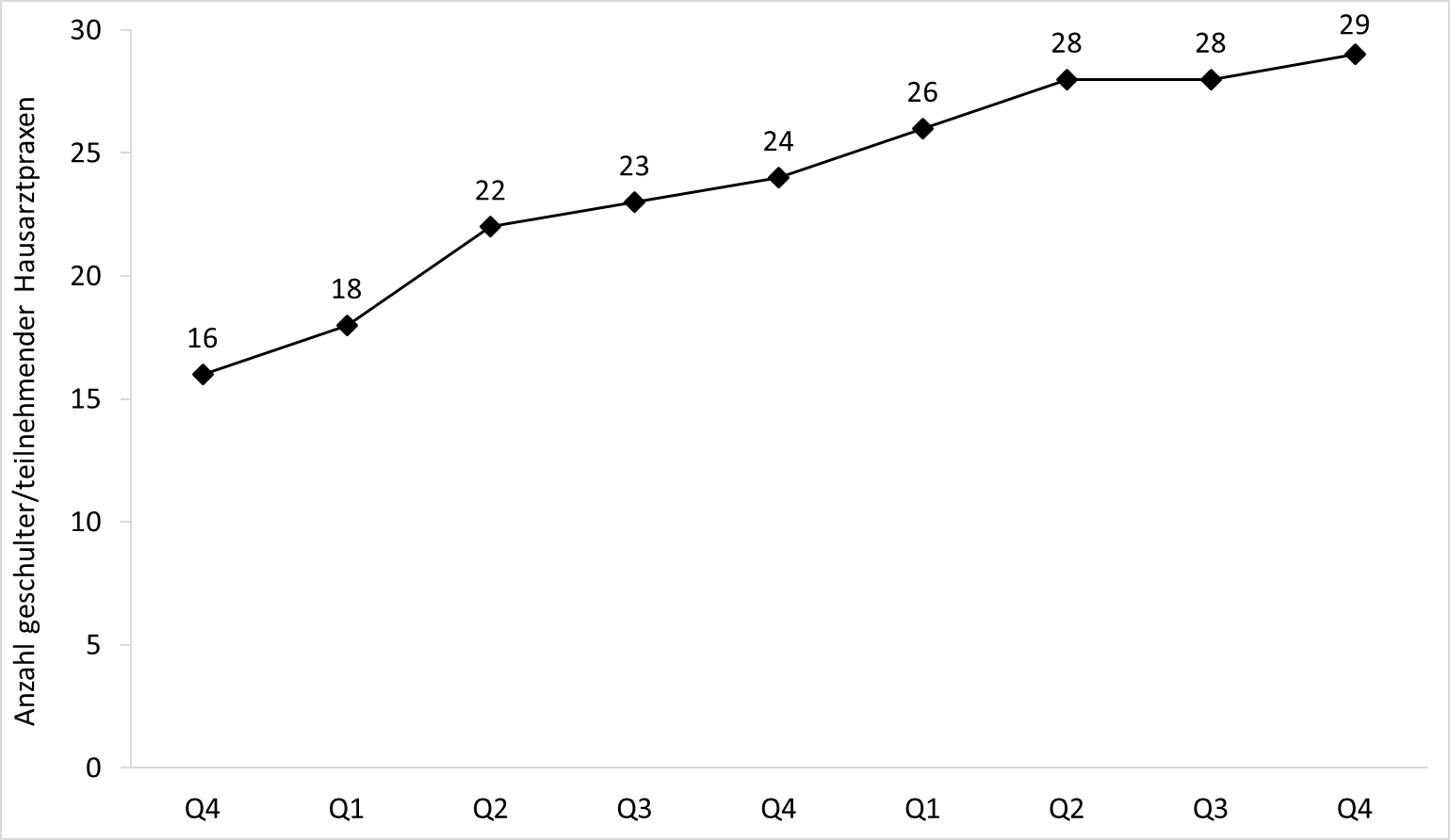


2018

2019

2017
