# Supplementary material for: Evaluation of satisfaction with and relief vs. workload by a general practitioner-centered dementia care project: early information and support in dementia (FIDEM) in Göttingen, Germany
Source: Nervenarzt. 2023 Oct 5;94(11):1034–42. [Article in German] doi: 10.1007/s00115-023-01557-6 (PMC10620282; doi:10.1007/s00115-023-01557-6)
Supplement: Supplementary file 2 [file 115_2023_1557_MOESM2_ESM.docx]

**Online Supplement**

**eTab. 1:** Formulierung der Items zur Evaluation von FIDEM

| *Item-Formulierungen* | *Antwortskala* |
| --- | --- |
| **(A1) Zufriedenheit mit FIDEM** |  |
| *Einleitung:* „Wie zufrieden sind Sie auf einer Skala von 0 („keine“) bis 10 („maximale Zufriedenheit“) mit folgenden Aspekten von FIDEM:“ |  |
| 1. Koordinationsebene | 11-stufig^1^ |
| 2. Vermittlungspfad |  |
| 3. Kooperations-/Netzwerkpartner |  |
| 4. Zufriedenheit mit der eigenen Beteiligung |  |
| 5. Zufriedenheit mit dem Projekt allgemein/insgesamt |  |
| **(A2) Arbeitszufriedenheit** |  |
| *Einleitung:* „Wie zufrieden sind Sie auf einer Skala von 0 („keine“) bis 10 („maximale Zufriedenheit“) mit folgenden Aspekten Ihrer Arbeit:“ |  |
| 1. Inhaltlich | 11-stufig^1^ |
| 2. Strukturell (Infrastruktur, Räumlichkeiten, Ausstattung etc.) |  |
| 3. Personell (Mitarbeiter) |  |
| 4. Kollegial (Austausch, Unterstützung, Qualitätszirkel etc.) |  |
| 5. Motivational (Projekte, Fortbildung, Zusatzqualifikationen etc.) |  |
| 6. Leistungsbezogen (eigene Leistung, Gesamtleistung der Praxis etc.) |  |
| 7. Arbeitszufriedenheit allgemein/insgesamt |  |
| **(B) Be-/Entlastung durch FIDEM** |  |
| *Einleitung:* „Meine Beteiligung als Kooperationspartner bedeutet für mich auf einer Skala von -5 bis +5…“ |  |
| 1. …zeitliche Be-/Entlastung | 11-stufig^2^ |
| 2. …strukturelle Be-/Entlastung (Infrastruktur, Räumlichkeiten, Ausstattung etc.) |  |
| 3. …personelle Be-/Entlastung (Mitarbeiter) |  |
| 4. …Be-/Entlastung durch FIDEM allgemein/insgesamt |  |

*Anmerkungen.* Alle in den Items formulierten Statements konnten auf 11-stufigen numerischen Skalen mit zwei Ankern von ^1^0 = „keine Zufriedenheit“ bis 10 = „maximale Zufriedenheit“, bzw. ^2^-5 = „extreme Belastung“ bis +5 = „extreme Entlastung“ bewertet werden.
